# Future steps in cardio-oncology—a national multidisciplinary survey among healthcare professionals in the Netherlands

**DOI:** 10.1007/s11764-022-01163-6

**Published:** 2022-02-04

**Authors:** Yvonne Koop, Arco J. Teske, Iris Wanders, Hanneke Meijer, J. H. A. M. (Hans) Kaanders, Olivier C. Manintveld, H. Carlijne Hassing, Hester Vermeulen, Angela H. E. M. Maas, Dick-Johan van Spronsen, Femke Atsma, Saloua El Messaoudi

**Affiliations:** 1 Department of Cardiology, Radboud University Medical Center, Nijmegen, The Netherlands; 2 Department of Cardiology, University Medical Center Utrecht, University of Utrecht, Utrecht, The Netherlands; 3 Department of Radiation Oncology, Radboud University Medical Center, Nijmegen, The Netherlands; 4 Department of Cardiology, Erasmus University Medical Center, Rotterdam, The Netherlands; 5 Scientific Institute for Quality of Healthcare, Radboud University Medical Center, Nijmegen, The Netherlands; 6 Faculty of Health and Social Studies, Research Department of Emergency and Critical Care, HAN University of Applied Sciences, Nijmegen, The Netherlands; 7 Department of Hematology, Radboud University Medical Center, Nijmegen, The Netherlands

**Keywords:** Cardio-oncology, Healthcare professionals, Survey, Guideline implementation, Quality of care, Cancer therapy–related cardiac dysfunction

## Abstract

**Background:**

The awareness of cancer therapy–related adverse cardiac effects is fueled by recent literature on cardiotoxicity incidence and detection strategies. Although this influences the sense of urgency, in current practice, cardiotoxicity monitoring and treatment is not structurally performed. With this study, we aimed to evaluate current perspectives on cardio-oncology and to assess needs, ultimately to determine an agenda for improvements in current practice.

**Material and methods:**

A national multidisciplinary 36-question survey was conducted. The survey was developed by a multidisciplinary team, theoretically based on an implementation checklist and distributed by email, through cardiology and oncology societies as well as social media.

**Results:**

One hundred ninety professionals completed the survey, of which 66 were cardiologists, 66 radiation oncologists, and 58 medical oncologists and hematologists. Many professionals were unaware of their specialisms’ cardio-oncology guidelines: 62.1% of cardiologists and 29.3% of the hematologists and medical oncologists respectively. Many cardiologists (*N* = 46; 69.7%), radiation oncologists (*N* = 45; 68.2%), and hematologists and medical oncologists (*N* = 38; 65.5%) expressed that they did not have sufficient knowledge to treat cardio-oncology patients and would either refer a patient or aspire to gain more knowledge on the topic.

**Conclusion:**

The field of cardio-oncology is advancing rapidly, with progress in stratification and detection strategies leading to the development of new guidelines and consensus statements. However, the application of these guidelines in current practice appears to be lagging. Professionals express a need for additional training and a practical guideline including risk stratification, monitoring, and treatment strategies. Multidisciplinary discussion and consensus on cardio-oncology care is vital to improve implementation of cardio-oncology guidelines, ultimately to improve cardiac care for oncology patients.

**Supplementary Information:**

The online version contains supplementary material available at 10.1007/s11764-022-01163-6.

## Background

Cardiovascular diseases (CVDs) and cancer are listed as the most common causes of death [1]. CVD constitute a common cause of morbidity and mortality in cancer survivors, which is partially explained by complications resulting from cancer therapy [2, 3]. Numerous cardiovascular toxicities are reported as a result of modern (targeted) therapies to improve cancer-related prognosis [4]. Cardiotoxic effects may accelerate the development of CVD, in particular when traditional CVD risk factors are present [5]. The increasing incidence of cancer therapy–related cardiac dysfunction (CTRCD) and increasing cancer survivorship have led to the emergence of cardio-oncology, in which prevention, patient-centered stratification, early detection, and monitoring of CTRCD are important pillars [6].

In recent years, research has increasingly focused on the association between cancer treatments and CVD resulting in an increase in awareness. Nevertheless, cardio-oncological care is often not a part of standard care yet, with overall low rates of cardiac care and a large heterogeneity between hospitals [7]. This variation in applied care might stem from the novelty of this field and gaps in current knowledge might complicate implementation in clinical care. For example, the absence of a validated risk stratification algorithm could lead to either over- or underutilization of care. This in turn leads to the occurrence of potentially preventable cardiac side effects in cancer patients or the unneeded interruption of essential cancer treatments [5]. To ensure continued delivery of healthcare improvements, it is crucial to have a unified view on CTRCD based on guidelines and to stimulate multidisciplinary collaboration. So far, the extent of healthcare utilization has been studied, but little is known about the views on implementation and application of cardio-oncology care [7, 8].

In order to define the necessary steps to move this field forward and identify gaps in the application of cardio-oncology knowledge, this study aims to get insight in professionals’ perspectives on this topic in the Netherlands.

## Material and methods

### Design and population

We conducted a multidisciplinary nationwide cross-sectional study in the Netherlands. Cardiologists, medical oncologists, hematologists, and radiation oncologists were invited for participation in this study and to fill out a 36-question survey between April and June 2021. The survey was conducted using Surveymonkey and distributed by email, using the newsletter of the “Netherlands comprehensive cancer organization,” the online environment of the “Dutch Society of Cardiology,” and social media platforms. The survey was sent by email to the medical secretary of the cardiology, oncology, hematology, and radiotherapy medical department of university and top-clinical hospitals; the secretary distributed the survey to all medical specialists at the department. All medical departments received an email reminder twice and one reminder was posted on social media. The primary aim was described at the start of the survey and consent was implied if the survey was completed. The Medical Research Ethics Committee of Arnhem-Nijmegen provided a waiver since the study did not require an ethical review. The study followed Dutch privacy requirements.

### Survey development

The survey is based on the seven domains that influence practice, as defined in the Tailored Implementation for Chronic Diseases (TICD) checklist (Table 1) [9]. A previous international cardio-oncology survey provided the basis for survey content [10]. A first draft was discussed by a multidisciplinary team of three cardiologists, one hematologist, two radiation oncologists, and two epidemiologists with cardio-oncology experience of the Radboud University Medical Center and the Utrecht University Medical Center. The survey was reviewed after a pilot among forty cardiologists in the region Arnhem-Nijmegen; the survey was adjusted to apply to oncologists, hematologists, and radiation oncologists, baseline questions were refined to ensure anonymity, and clinical cases were added to replace knowledge-related questions. The final version consisted of 36 questions: eight demographic and current practice questions, eight 5-point Likert scale questions, 19 multiple-choice questions, and one open-ended question. The 5-point Likert scale questions ranged from completely disagree to completely agree. All questions were divided in 7 categories. The survey’s first category evaluated the perceptions of cardio-oncology, related to the primary aim of cardio-oncology care and the perceived relevance of monitoring. The second category focused on cardiotoxicity, more specifically the definition, risk, and incidence. The third category contained two cardio-oncology clinical cases. Category 4 assessed whether professionals were aware of guidelines and clinical protocols and if additional training is necessary. The fifth category evaluated patient factors, focusing on the relevance to discuss potential cardiotoxic effects with patients. Category six evaluated cardio-oncology collaborations and category 7 reviewed improvements regarding cardio-oncology organization.

**Table 1:** TICD checklist domains and survey categories

	Survey category
Guideline factors Quality, accessibility, feasibility, and practice	Guidelines and training Cardiotoxicity
Individual health professional factors Knowledge, attitude, and professional behavior	Cardio-oncology perceptions Cardiotoxicity Clinical cases Multidisciplinary collaboration
Patient factors Patient needs, knowledge, and preferences	Patient factors
Professional interactions Communication, influence, team, and referral processes	Multidisciplinary collaboration Organizational improvements
Incentives and resources Availability of resources, incentives, and quality assurance systems	Multidisciplinary collaboration
Capacity for organizational change Authority, leadership, regulations, and priority of change	Organizational improvements
Social, political and legal factors Individual influence, contracts, and funding	Organizational improvements

*TICD* tailored implementation for chronic diseases [9]

### Data analysis

Demographic and current practice data were normally distributed and described with mean, and standard deviation or proportions were used where appropriate. Results are based on survey responses on each separate question using descriptive data per subgroup: radiation oncologists, cardiologists, medical oncologists, and hematologists (Supplementary Table 1). With subgroup data, differences in perceptions between specialisms were assessed. Response rates were not calculated, because the survey was openly distributed. Statistical analysis was supported by R Statistical Software (version 3.6.2., R Foundation).

## Results

### Population

A total of 190 professionals completed the survey, 66 (34.7%) were cardiologists, 66 (34.7%) were radiation oncologists, 29 (15.3%) were hematologists, and 29 (15.3%) were medical oncologist (Table 2). Most respondents were employed at a university medical center (*N* = 96; 50.5%), the majority completed a PhD trajectory (*N* = 115; 60.2%), and the average work experience was 12.2 years (± SD 9.1). As stated by the professionals, in 75 cases, a cardio-oncology unit was present at their hospital with an average of 1–10 new cardio-oncology patients per month. Related to privacy regulations, it is unclear how many professionals were employed by the same hospital.

**Table 2:** Demographic data of respondents

	Healthcare professionals (*N* = 190)
Sex (*N*, %)	
Female	99 (52)
Male	91 (48)
Specialism (*N*, %)	
Cardiologist^1^	66 (35)
Intervention	16
Imaging	23
Heart failure	12
Electrophysiology and devices	8
Other	9
Medical oncologist	29 (15)
Breast cancer	10
Gynecological	4
Urologic	4
Palliative care	4
Lung	2
Radiation oncologist	66 (35)
Hematologist	29 (15)
Work experience in years, median (IQR)	10 (5–18.8)
Type of hospital (*N*, %)	
University medical center	96 (51)
Top-clinical hospital^2^	70 (37)
General hospital Private hospital	22 (12) 2 (1)
Cardio-oncology unit at hospital (*N*, %)	
Yes	75 (40)
No	115 (60)
Completed PhD trajectory (*N*, %)	
Yes	115 (60)
No	75 (40)^4^
Number of new cardio-oncology patients at outpatient ward per month (*N*, %)^3^	
None	18 (10)
1–10	123 (65)
11–20	37 (20)
21–30	9 (5)
≥ 30	3 (2)

^1^Only certified subspecialties are provided, other non-certified subspecialties for cardiologists are devices, cardio-oncology, congenital heart disease, and cardiovascular disease in women

^2^A top-clinical hospital is a secondary care setting; these hospitals provide both basic and complex care

^3^Cardio-oncology patients were defined as oncology patients with traditional cardiovascular risk factors receiving cardiotoxic treatment or oncology patients with pre-existing cardiovascular disease

^4^15 respondents were PhD candidates at the time of the study

### Cardio-oncology perception and definitions

**Fig. 1:**
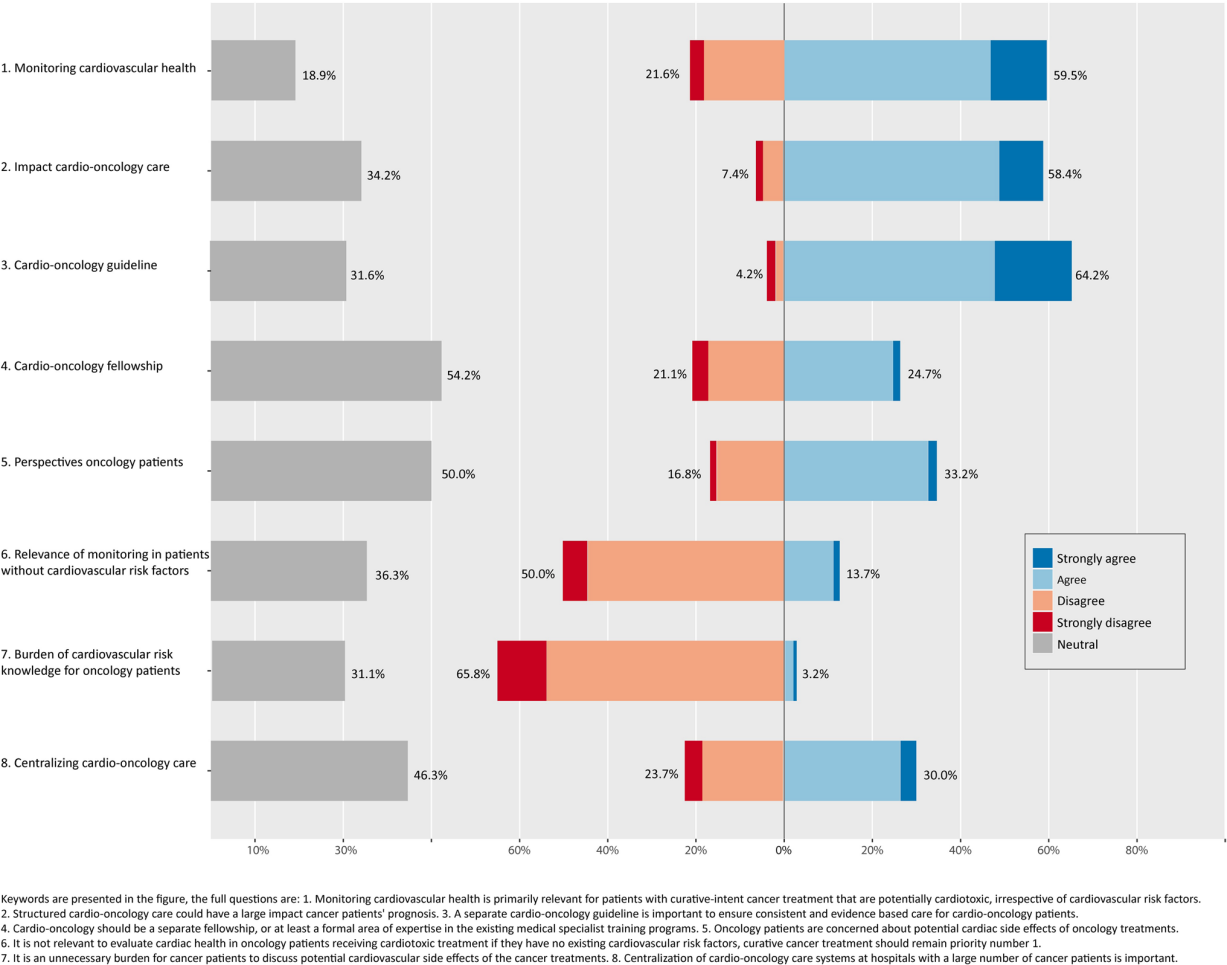
Overview of the 5-point Likert scale questions

xOf all professionals, 63 (33.2%) responded that patients are concerned about potential cardiotoxic effects of cancer treatment (Fig. 1). In line with this figure, 83 (43.7%) professionals chose patient education as a goal of cardio-oncology care, most of which were cardiologists (*N* = 33; 50%) and radiation oncologists (*N* = 30; 45.5%), compared to eight medical oncologists (27.6%).

The main goals of cardio-oncology are risk stratification, diagnosing, monitoring, and mitigating cardiotoxic side effects, as selected by the respondents. The majority of professionals (*N* = 166; 87.4%) perceived monitoring cardiovascular side effects after and during cancer treatment as a relevant strategy to improve patients’ long-term health.

### Current practice and collaboration

The cardiotoxicity definition “a reduction of left ventricular ejection fraction (LVEF) > 10% to value < 53% or a relative global longitudinal strain (GLS) reduction of 10% to a value <  − 19%” was mainly used by cardiologists (*N* = 23; 34.8%). Of all professionals, 21 cardiologists (31.8%), 43 radiation oncologists (65.2%), and 35 hematologists or medical oncologists (60.3%) stated either they were unaware of the definition in their hospital or there was no clear definition.

The most commonly used screening methods for cardiotoxicity were MUGA scan (34.2%), 2D-echocardiography (42.1%), and an ECG (32.1%), as a more ideal screening tool advanced echocardiography (29.5%) and CMR (17.4%) were selected. With MUGA patients repeatedly exposed to radiation, the recommended standard screening tool in guidelines is echocardiography [11, 12]. Seventeen (25.8%) cardiologists organize cardio-oncology care in their hospital with regular multidisciplinary meetings, compared to five (8.6%) hematologists and medical oncologists and one (1.5%) radiation oncologist.

### Clinical cases

Two clinical cases were described; in the first case, a 41-year-old woman diagnosed with breast cancer was treated with four cycles of doxorubicin and cyclophosphamide followed by 1-year trastuzumab treatment. After 6 months, LVEF declined from 58% at baseline to 45%.

Literature recommends to treat asymptomatic LVEF decline during trastuzumab treatment with appropriate cardiac medications and monitor LVEF during follow-up [13, 14]. Only twelve (18.2%) cardiologists, one (1.7%) hematologist, no medical oncologists, and two (3%) radiation oncologists selected this answer. The most selected response was interruption of trastuzumab and restart after LVEF improvement with cardiac medication (*N* = 69; 36.3%). Premature interruption of trastuzumab however increases the number of breast cancer recurrences significantly, and therefore according to current recommendations continuation of trastuzumab combined with the start of heart failure therapy is preferred [15].

The second case described a 62-year-old man with an upper-gastrointestinal carcinoma who received three neo-adjuvant cycles and three adjuvant cycles of epirubicin, cisplatin, and capecitabine. After the first adjuvant cycle, the patient experiences chest pain with exacerbation during exercise. Evidence on capecitabine cardiotoxicity treatment is scarce, though discontinuation of capecitabine is recommended to relieve and prevent future symptoms of dyspnea and chest pain [16]. In line with this recommendation, a minority of 25 (37.9%) cardiologists, 24 (36.4%) radiation oncologists, 9 (31.0%) hematologists, and a small majority of 16 (55.2%) medical oncologists would (recommend to) discontinue capecitabine or discuss with the treating-oncologist to switch capecitabine for a less cardiotoxic antimetabolite. Cardiologists and radiotherapists would primarily have the role of consultant in this situation. Though the majority of all professionals deviated from current recommendations and would continue capecitabine treatment. A total of 26 professionals (13.7%)—of which 20 were cardiologists—would continue capecitabine combined with a calcium channel blocker. In non-chemotherapy-related chest pain, this would be an appropriate choice, but this is rarely successful in capecitabine-related chest pain [16, 17].

### Guidelines and training

Of the cardiologists, 41 (62.1%) were aware of the ESC cardio-oncology position paper and 15 (22.7%) of the multimodality imaging consensus statement of the European and American imaging associations, and 36 (54.5%) use these guidelines in current practice [3, 11]. The ESMO guidelines were known by five hematologists (17.2%), twelve oncologists (41.4%), and three radiation oncologists (4.5%); respectively, five (17.2%), seven (24.1%), and two (3%) use this guideline in current practice [18, 19].

Cardio-oncology knowledge was primarily acquired during their medical specialist training period (*N* = 83; 43.7%) by all professionals. Especially cardiologists (*N* = 33; 50.5%) and medical oncologists (*N* = 13; 44.8%) used scientific literature to improve knowledge. Twelve cardiologists (18.2%), two hematologists (6.9%), ten medical oncologists (34.5%), and eight radiation oncologists (12.1%) stated to have sufficient knowledge on cardiotoxic effects of cancer therapy in clinical practice. Additional educational activities on this topic should include recommendations from guidelines (*N* = 92; 48.4%) and clinical scenario training (*N* = 44; 23.2%). A national cardio-oncology guideline was selected by 87 (45.8%) professionals as an important tool for improving current practice, specifying risk stratification, monitoring, and treatment of cardiotoxicity.

## Discussion

Cardio-oncology is rapidly evolving with a continuing increase in initiatives to study and improve cardiac care for cancer patients. Multidisciplinary collaborations to share knowledge is a constant and pivotal recommendation in organizing cardio-oncological care. This national survey aimed to evaluate perceptions of all involved disciplines, showing that diagnosing, monitoring, and treating cardiotoxic effects were overall perceived as important goals of cardio-oncology. Although education is important for patients to understand the potential cardiac effects and to actively participate in long-term surveillance with early recognition of symptoms, education of patients was in general not perceived as a goal of cardio-oncology by the medical specialists [20].

Remarkably, cardio-oncology guidelines were not widely known or used in clinical practice. Our findings are in line with previous international surveys which also show a discrepancy between guideline recommendations and implementation in clinical practice [10, 21]. With recent advancements in (inter)national educational activities via conferences, consensus statements, councils, and working groups of various cardiology and oncology specialty societies, our initial expectation was that guideline knowledge would have improved in the past years. Neither ESMO nor ESC guidelines were widely known or applied. Our data suggests that engaging professionals without prior cardio-oncology experience seems to be a challenge not easily solved with large-scale educational activities. As previously suggested by the international CardioOncology society (ICOS) and the Canadian cardiac oncology network (CCON), a formal structure to train professionals should be established to enhance professionals’ expertise on cardiovascular care in cancer patients [22]. This highlights the importance of a cardio-oncology team recognized by all involved disciplines, centralized in hospitals specialized in cancer care. This would pave the way for a formal training program during professionals’ specialist training to enhance knowledge of all involved disciplines as well as ensuring agreeable protocols are established.

Professionals in this study shared the opinion that cardio-oncology care could significantly improve long-term prognosis of cancer patients and monitoring would be relevant for all patients receiving cardiotoxic cancer treatment, as opposed to a previous study in which cardiologists and oncologists did not agree on this topic [10]. Even though participants between the survey studies differed, this result could suggest an increase in awareness on cardiotoxic effects and the relevance of cardio-oncological care.

Due to the novelty of this subspecialty, the current level of scientific evidence leaves many uncertainties. The definitions of cardiotoxicity continue to differ between studies, and treatment of cardiotoxicity is largely based on heart failure trials where cancer patients were typically excluded [23, 24]. This results in knowledge gaps related to risk stratification, monitoring, and treatment. Cardiac care in cancer patients is complex as it requires basic understanding of oncologic pharmacology, cardiotoxicity risk factors, and pathophysiology of common cardiovascular diseases, as well as early detection strategies and cardioprotective treatment options. These factors, combined with differences in guideline recommendations, significantly complicate the implementation in clinical care. Differences in recommendations might also result in varying perceptions on cardiac care in cancer patients between disciplines. Current recommendations in the recent ESMO consensus statement and ESC heart failure guideline include baseline risk stratification to determine the monitoring type, frequency, and timing [19, 25], although with ESMO the monitoring schedule is determined using baseline ejection faction, as opposed to the ESC guidelines in which baseline CV risk assessment using the HFA-ICOS risk assessment is recommended [19, 25]. Indeed, establishing a baseline LVEF in all cancer patients would not be feasible due to the sheer number of cancer patients treated with potential cardiotoxic systemic anti-cancer medication. This is the main reason why a baseline risk assessment is advised by the HFA-ICOS in order to prevent unnecessary diagnostics in low-risk patients and to make optimal use of limited medical resources such as echocardiography.

**Fig. 2:**
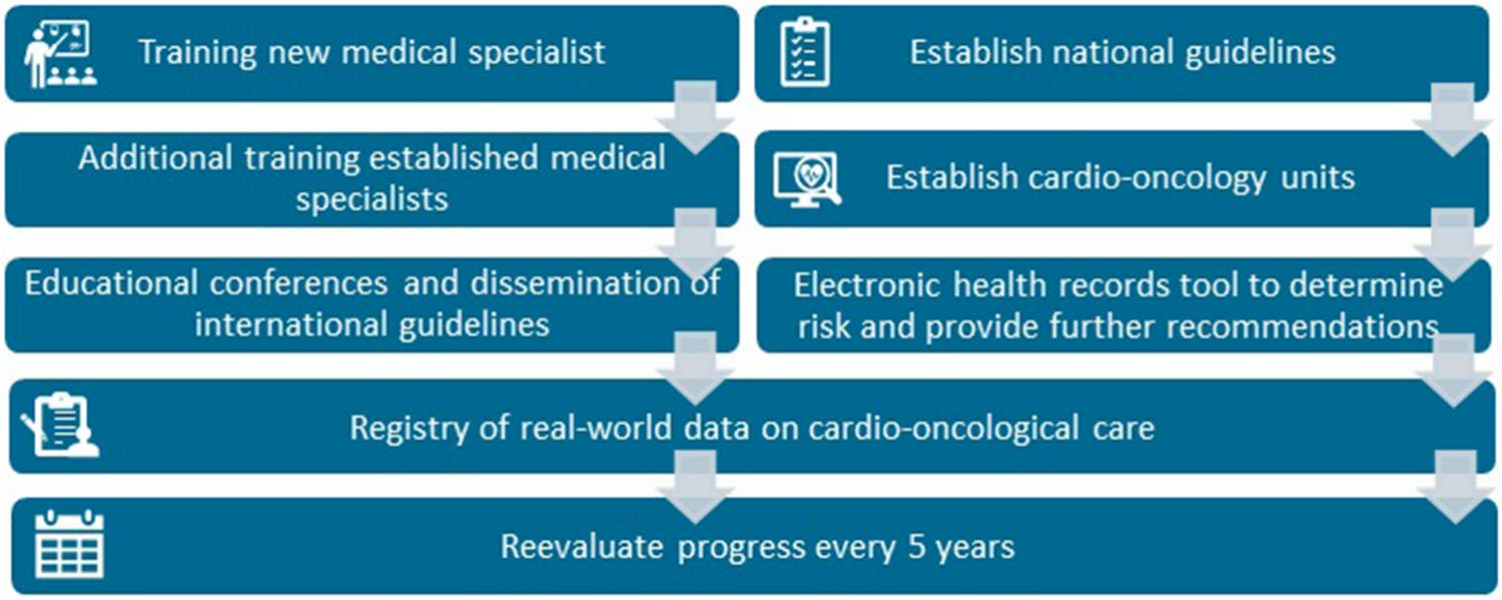
Overview of recommendations to improve cardio-oncology care

Still, both research and clinical collaborations between disciplines are increasing, and an outline for training future professionals in cardio-oncology has been developed [22]. These collaborations should also result in a cardio-oncology guideline developed and acknowledged by all involved disciplines. In our opinion training, collaboration and uniform recommendations on risk stratification, monitoring, and treatment are fundamental for successful implementation of cardio-oncological care throughout the involved spectrum of relevant disciplines concerned with the treatment of cancer patients in clinical practice (Fig. 2). However, due to the complexity of cardio-oncological care, this should be centralized in oncological centers with a dedicated team for diagnosis and treatment of cardiotoxicity. All involved disciplines should be trained in early recognition of CTRCD symptoms. Finally, real-world data on implementation of screening and treatment of cardiotoxicity should be evaluated in order to establish the effectivity of the aforementioned developments in this field. Recently, a national cardio-oncology registry (ONCOR) has been launched which could potentially address such questions [PMID 33201485]. Such a registry could also validate proposed risk assessment tools, disparities in cardio-oncology, and also the change in management and its impact in oncological care throughout the years.

### Limitations

A large proportion of professionals was employed by a university hospital and completed a PhD, suggesting the sample is not fully representative for all medical specialists in the Netherlands. Professionals who are aware of cardiotoxicity risks might be more inclined to complete the survey. In addition, due to privacy regulations, it is unclear which professionals were employed by the same hospital. Therefore, the study results could reflect an overestimation of knowledge and collaboration. Exact response rates could not be calculated, and the survey was openly distributed; therefore, it is unclear how many professionals received it. The number of medical professionals with a registration in cardiology, radiotherapy, or internal medicine (e.g., medical oncologist or hematologists) is 945, 185, and 1980, respectively. However, calculating response rates with these numbers would likely be a large underestimation of the actual response rate, because these numbers are based on all professionals registered in the Netherlands and therefore include professionals registered but not currently employed as well as retired professionals who maintained a registration in their field. In addition, internal medicine is an even larger overestimation of the number of hematologists and medical oncologists, because the large specialization also includes nephrology, vascular medicine, and infectious diseases. Therefore, response rates cannot be presented.

### Conclusion

The field of cardio-oncology is advancing rapidly, with progress in stratification and detection strategies leading to the development of new guidelines, position papers, and consensus statements. Our survey showed that the application of the available recommendations in current practice seems to be lagging and that there is a clear need for additional training, both clinical training and online training tools, as well as structured multidisciplinary collaboration and a national guidelines based on international recommendations. Further improvement in this field is warranted by formal education, multidisciplinary collaboration, centralization of cardio-oncology care, and national guidelines with practical recommendations. In addition, a standardized tool for cardio-oncological care with risk stratification and monitoring recommendations in electronic health records could provide the infrastructure to advance current cardio-oncological care and collaboration.

## Supplementary Information

Below is the link to the electronic supplementary material.

Supplementary file1 (PDF 235 KB)

## Data Availability

The datasets generated during and/or analyzed during the current study are available from the corresponding author on reasonable request.
