# Supplementary material for: Future steps in cardio-oncology—a national multidisciplinary survey among healthcare professionals in the Netherlands
Source: J Cancer Surviv. 2022 Feb 4;17(4):1131–8. doi: 10.1007/s11764-022-01163-6 (PMC10285002; doi:10.1007/s11764-022-01163-6)
Supplement: Supplementary file 1 — Supplementary file1 (PDF 235 KB) [file 11764_2022_1163_MOESM1_ESM.pdf]

Supplementary table 1. Survey results multiple choice questions

	Cardiologist (N=66)	Hematologist (N=29)	Oncologist (N=29)	Radiotherapist (N=66)	Total (N=190)
1. Gender					
Male	42 (63.6%)	12 (41.4%)	9 (31.0%)	28 (42.4%)	91 (47.9%)
Female	24 (36.4%)	17 (58.6%)	20 (69.0%)	38 (57.6%)	99 (52.1%)
2. Type of hospital					
General hospital	7 (10.6%)	0 (0.0%)	0 (0.0%)	15 (22.7%)	22 (11.6%)
Private clinic	0 (0.0%)	0 (0.0%)	0 (0.0%)	2 (3.0%)	2 (1.1%)
Topclinical hospital	34 (51.5%)	13 (44.8%)	9 (31.0%)	14 (21.2%)	70 (36.8%)
University medical center	25 (37.9%)	16 (55.2%)	20 (69.0%)	35 (53.0%)	96 (50.5%)
3. Years of experience as a medical specialist, median (IQR)	9.5 (4.2, 14.8)	10.0 (5.0, 20.0)	10.0 (4.0, 20.0)	12.5 (5.2, 21.0)	10.0 (5.0, 18.8)
4. Did you finish a PhD?					
Yes	41 (62.1%)	20 (69.0%)	20 (69.0%)	34 (51.5%)	115 (60.5%)
No, but I am currently working on my PhD dissertation	7 (10.6%)	4 (13.8%)	2 (6.9%)	2 (3.0%)	15 (7.9%)
No	18 (27.3%)	5 (17.2%)	7 (24.1%)	30 (45.5%)	60 (31.6%)
5. Is there a multidisciplinary cardio oncology clinic in your hospital?					
Yes	31 (47.0%)	13 (44.8%)	12 (41.4%)	19 (28.8%)	75 (39.5%)
No	35 (53.0%)	16 (55.2%)	17 (58.6%)	47 (71.2%)	115 (60.5%)
6. How many new cardio-oncology patients do you treat per month?					
Geen	13 (19.7%)	0 (0.0%)	1 (3.4%)	4 (6.1%)	18 (9.5%)
1-10	40 (60.6%)	20 (69.0%)	18 (62.1%)	45 (68.2%)	123 (64.7%)
11-20	10 (15.2%)	6 (20.7%)	8 (27.6%)	13 (19.7%)	37 (19.5%)
21-30	2 (3.0%)	1 (3.4%)	2 (6.9%)	4 (6.1%)	9 (4.7%)
> 30	1 (1.5%)	2 (6.9%)	0 (0.0%)	0 (0.0%)	3 (1.6%)
7. What is an important aim of the subspecialism cardio-oncology? (select all that apply)					
Diagnosing cardiotoxic side effects of cancer therapy	46 (69.7%)	27 (93.1%)	20 (69.0%)	38 (57.6%)	131 (68.9%)
Monitoring and treating cancer patients with cancer therapy related cardiac dysfunction (CTRCD)	51 (77.3%)	28 (96.6%)	22 (75.9%)	47 (71.2%)	148 (77.9%)
Recognizing / risk stratification of patients with a high cardiotoxicity risk and minimize this risk with therapy recommendations	45 (68.2%)	26 (89.7%)	17 (58.6%)	43 (65.2%)	131 (68.9%)
Follow-up of cancer survivors if they received cardiotoxic treatment to screen for symptoms of cardiac dysfunction	45 (68.2%)	25 (86.2%)	10 (34.5%)	42 (63.6%)	122 (64.2%)
Educating patients on the potential cardiotoxic effects of cancer therapy and on recognizing symptoms	33 (50.0%)	12 (41.4%)	8 (27.6%)	30 (45.5%)	83 (43.7%)
8. Is, in your opinion, monitoring for cardiotoxic side effects of cancer therapy relevant for the long-term health of cancer patients?					
Missing	7 (10.6%)	0 (0.0%)	4 (13.8%)	8 (12.1%)	19 (10.0%)
Yes, for all cancer treatments	7 (10.6%)	6 (20.7%)	1 (3.4%)	1 (1.5%)	15 (7.9%)
Yes, but only for specific cardiotoxic treatments regardless of cardiovascular health	42 (63.6%)	20 (69.0%)	17 (58.6%)	42 (63.6%)	121 (63.7%)
Yes, but only for specific cardiotoxic treatments in cancer patients with pre-existing cardiovascular diseases or cardiac risk factors	8 (12.1%)	2 (6.9%)	6 (20.7%)	14 (21.2%)	30 (15.8%)
No, incidence of cardiotoxic side effects is very low	0 (0.0%)	0 (0.0%)	0 (0.0%)	1 (1.5%)	1 (0.5%)
No, monitoring is not relevant since there is no guideline for therapeutic policy if cardiotoxicity is detected	2 (3.0%)	1 (3.4%)	1 (3.4%)	0 (0.0%)	4 (2.1%)
9. In your hospital cardiotoxicty is defined as:					
NA/Missing	8 (12.1%)	1 (3.4%)	5 (17.2%)	14 (21.2%)	28 (14.7%)
Functional or structural cardiac damage related to cancer treatment	12 (18.2%)	7 (24.1%)	5 (17.2%)	9 (13.6%)	33 (17.4%)
An absolute LVEF reduction of more than 10% to a value below 53% or a relative GLS reduction of more than 11% to a value below -19%	23 (34.8%)	2 (6.9%)	3 (10.3%)	0 (0.0%)	28 (14.7%)
There is no clear definition	8 (12.1%)	8 (27.6%)	7 (24.1%)	10 (15.2%)	33 (17.4%)
I don't know if there is a clear definition in our hospital	13 (19.7%)	11 (37.9%)	9 (31.0%)	33 (50.0%)	66 (34.7%)
Other, namely..	2 (3.0%)	0 (0.0%)	0 (0.0%)	0 (0.0%)	2 (1.1%)
	Other: An absolute LVEF reduction of more than 10% to a value below 53% or a relative GLS reduction of more than 15% to a value below -19%, An absolute LVEF reduction of more than 10% or an LVEF below 45%				
10. Which screening tools do you currently use to detect cardiotoxicity? (select all that apply)					
MUGA (LVEF)	24 (36.4%)	15 (51.7%)	20 (69.0%)	6 (9.1%)	65 (34.2%)
2D echocardiography (LVEF)	36 (54.5%)	22 (75.9%)	16 (55.2%)	6 (9.1%)	80 (42.1%)
3D advanced echocardiography (GLS and LVEF)	32 (48.5%)	5 (17.2%)	3 (10.3%)	5 (7.6%)	45 (23.7%)
CMR with mapping sequences	13 (19.7%)	4 (13.8%)	2 (6.9%)	1 (1.5%)	20 (10.5%)
Cardiac biomarker testing (NT-proBNP or Troponin)	17 (25.8%)	19 (65.5%)	6 (20.7%)	7 (10.6%)	49 (25.8%)
ECG	29 (43.9%)	13 (44.8%)	14 (48.3%)	5 (7.6%)	61 (32.1%)
I don't know which tools are currently used	1 (1.5%)	2 (6.9%)	1 (3.4%)	39 (59.1%)	43 (22.6%)
11. Which screening tools would you ideally use to detect cardiotoxicity? (select all that apply)					
MUGA (LVEF)	8 (12.1%)	6 (20.7%)	5 (17.2%)	2 (3.0%)	21 (11.1%)
2D echocardiography (LVEF)	21 (31.8%)	13 (44.8%)	12 (41.4%)	4 (6.1%)	50 (26.3%)
3D advanced echocardiography (GLS and LVEF)	40 (60.6%)	7 (24.1%)	3 (10.3%)	6 (9.1%)	56 (29.5%)
CMR with mapping sequences	25 (37.9%)	3 (10.3%)	2 (6.9%)	3 (4.5%)	33 (17.4%)
Cardiac biomarker testing (NT-proBNP or Troponin)	21 (31.8%)	8 (27.6%)	6 (20.7%)	4 (6.1%)	39 (20.5%)
ECG	18 (27.3%)	5 (17.2%)	5 (17.2%)	4 (6.1%)	32 (16.8%)
I don't know	1 (1.5%)	9 (31.0%)	8 (27.6%)	41 (62.1%)	59 (31.1%)
12. Based on your clinical expertise, when are cancer patients receiving chemotherapy (anthracyclines) at highest risk to develop cancer therapy related cardiac dysfunction?					
Missing	8 (12.1%)	1 (3.4%)	5 (17.2%)	14 (21.2%)	28 (14.7%)
During cancer treatment (active treatment period)	26 (39.4%)	7 (24.1%)	3 (10.3%)	4 (6.1%)	40 (21.1%)
Within 1 year after cancer treatment (short-term risk)	18 (27.3%)	11 (37.9%)	3 (10.3%)	15 (22.7%)	47 (24.7%)
Between 1 and 5 years after cancer treatment	10 (15.2%)	7 (24.1%)	7 (24.1%)	20 (30.3%)	44 (23.2%)
More than 5 years after cancer treatment (long-term risk)	4 (6.1%)	3 (10.3%)	11 (37.9%)	13 (19.7%)	31 (16.3%)
13. Based on your clinical expertise, when are cancer patients receiving thoracic radiotherapy at highest risk to develop cancer therapy related cardiac dysfunction?					
Missing	8 (12.1%)	1 (3.4%)	5 (17.2%)	14 (21.2%)	28 (14.7%)
During cancer treatment (active treatment period)	1 (1.5%)	0 (0.0%)	0 (0.0%)	0 (0.0%)	1 (0.5%)
Within 1 year after cancer treatment (short-term risk)	4 (6.1%)	1 (3.4%)	2 (6.9%)	2 (3.0%)	9 (4.7%)
Between 1 and 5 years after cancer treatment	14 (21.2%)	8 (27.6%)	9 (31.0%)	11 (16.7%)	42 (22.1%)
More than 5 years after cancer treatment (long-term risk)	39 (59.1%)	19 (65.5%)	13 (44.8%)	39 (59.1%)	110 (57.9%)
14. What is in your opinion an acceptable risk for cardiotoxicity in cancer patients with curative-intent treatment					
Missing	8 (12.1%)	1 (3.4%)	5 (17.2%)	14 (21.2%)	28 (14.7%)
< 1%	3 (4.5%)	1 (3.4%)	2 (6.9%)	0 (0.0%)	6 (3.2%)
1 – 5%	13 (19.7%)	8 (27.6%)	6 (20.7%)	11 (16.7%)	38 (20.0%)
6 – 10%	7 (10.6%)	3 (10.3%)	0 (0.0%)	1 (1.5%)	11 (5.8%)
11 – 15%	2 (3.0%)	0 (0.0%)	0 (0.0%)	0 (0.0%)	2 (1.1%)
This depends, if a cancer treatment strongly improves cancer prognosis I accept a higher cardiotoxicity risk	33 (50.0%)	16 (55.2%)	16 (55.2%)	40 (60.6%)	105 (55.2%)
15. Clinical case 1 - What would be your treatment strategy? A 41-year old woman diagnosed with breast cancer was treated with 4 cycles of doxorubicin and cyclophosphamide followed by 1 year trastuzumab treatment. After 6 months LVEF declined from 58% at baseline to 45%.					
Missing	10 (15.2%)	11 (37.9%)	7 (24.1%)	36 (54.5%)	64 (33.7%)
Advise to stop trastuzumab treatment without a potential restart	1 (1.5%)	2 (6.9%)	3 (10.3%)	8 (12.1%)	14 (7.4%)
Interrupt trastuzumab treatment and wait for LVEF to recover before restart	4 (6.1%)	3 (10.3%)	9 (31.0%)	12 (18.2%)	28 (14.7%)
Interrupt trastuzumab, start cardiac medication and restart trastuzumab after LVEF recovery	39 (59.1%)	12 (41.4%)	10 (34.5%)	8 (12.1%)	69 (36.3%)
Continue trastuzumab treatment combined with cardiac medication (ACEi, betablocker)	12 (18.2%)	1 (3.4%)	0 (0.0%)	2 (3.0%)	15 (7.9%)
16. Clinical case 2 - What would be your treatment strategy? A 62-year old man with an upper-gastrointestinal carcinoma who received 3 neo-adjuvant cycles and 3 adjuvant cycles of epirubicin, cisplatin and capecitabine. After the first adjuvant cycle the patient experiences chest pain with exacerbation during exercise.					
Missing	9 (13.6%)	10 (34.5%)	7 (24.1%)	33 (50.0%)	59 (31.1%)
Continue chemotherapy after ruling out coronary artery disease	7 (10.6%)	9 (31.0%)	1 (3.4%)	6 (9.1%)	23 (12.1%)
Continue chemotherapy after ruling out coronary artery disease, and start a short-acting nitrate	5 (7.6%)	0 (0.0%)	0 (0.0%)	3 (4.5%)	8 (4.2%)
Continue chemotherapy after ruling out coronary artery disease, and start a calcium channel blocker	20 (30.3%)	1 (3.4%)	5 (17.2%)	0 (0.0%)	26 (13.7%)
Consult an oncologist to swith capecitabine for a less cardiotoxic antimetabolite	24 (36.4%)	6 (20.7%)	12 (41.4%)	18 (27.3%)	60 (31.6%)
Advise to stop chemotherapy without a potential restart	1 (1.5%)	3 (10.3%)	4 (13.8%)	6 (9.1%)	14 (7.4%)
17. Do you have sufficient knowledge on cardiotoxic effects of cancer therapy? (Select all that apply)					
Yes, I have sufficient knowledge which I could apply in clinical practice	12 (18.2%)	2 (6.9%)	10 (34.5%)	4 (6.1%)	28 (14.7%)
Yes, I treat patients with cardiotoxicty regularly	13 (19.7%)	3 (10.3%)	5 (17.2%)	2 (3.0%)	23 (12.1%)
If I needed to apply this knowledge in clinical practice then I would need additional training	30 (45.5%)	14 (48.3%)	8 (27.6%)	19 (28.8%)	71 (37.4%)
No, I would consult or refer the patiënt to a colleague	16 (24.2%)	8 (27.6%)	8 (27.6%)	26 (39.4%)	58 (30.5%)
18. Where did you acquire cardio-oncology knowledge? (select all that apply)					
During the medicine study	3 (4.5%)	5 (17.2%)	4 (13.8%)	8 (12.1%)	20 (10.5%)
During medical specialist training	27 (40.9%)	15 (51.7%)	17 (58.6%)	24 (36.4%)	83 (43.7%)
Congress	16 (24.2%)	8 (27.6%)	5 (17.2%)	13 (19.7%)	42 (22.1%)
Educational conference	29 (43.9%)	7 (24.1%)	7 (24.1%)	10 (15.2%)	53 (27.9%)
I don't have specific cardio-oncology knowledge	7 (10.6%)	2 (6.9%)	1 (3.4%)	10 (15.2%)	20 (10.5%)
Self study and / or scientific literature	33 (50.0%)	10 (34.5%)	13 (44.8%)	18 (27.3%)	74 (38.9%)
19. Are you familiar with a guideline regarding cardio-oncological care? (select all that apply)					
Yes, ESC position paper	41 (62.1%)	2 (6.9%)	3 (10.3%)	0 (0.0%)	46 (24.2%)
Yes, EACVI expert consensus	15 (22.7%)	0 (0.0%)	0 (0.0%)	0 (0.0%)	15 (7.9%)
Yes, ESMO guideline and/or consensus recommendation	4 (6.1%)	5 (17.2%)	12 (41.4%)	3 (4.5%)	24 (12.6%)
No, I am not familiar with any guidelines including cardio-oncological care	14 (21.2%)	15 (51.7%)	8 (27.6%)	39 (59.1%)	76 (40.0%)
No, currently there is no clear and practical guideline	2 (3.0%)	3 (10.3%)	3 (10.3%)	1 (1.5%)	9 (4.7%)
20. Do you use guideline recommendations in clinical practice?					
Missing	13 (19.7%)	10 (34.5%)	14 (48.3%)	43 (65.2%)	80 (42.1%)
Yes	36 (54.5%)	5 (17.2%)	7 (24.1%)	2 (3.0%)	50 (26.3%)
No	17 (25.8%)	14 (48.3%)	8 (27.6%)	21 (31.8%)	60 (31.6%)

21. Would you like additional training on cardio-oncological care? (select all that apply)					
Yes, especially examples from clinical practice	23 (34.8%)	3 (10.3%)	9 (31.0%)	9 (13.6%)	44 (23.2%)
Yes, especially regarding current guidelines for this population	40 (60.6%)	13 (44.8%)	15 (51.7%)	24 (36.4%)	92 (48.4%)
Yes, training regarding cardio-oncological care should be a part of the medical specialist training programme	25 (37.9%)	11 (37.9%)	10 (34.5%)	13 (19.7%)	59 (31.1%)
No, we have a cardio-oncologist who monitors and treats these patients	3 (4.5%)	4 (13.8%)	1 (3.4%)	3 (4.5%)	11 (5.8%)
No, I have sufficient knowledge	2 (3.0%)	0 (0.0%)	0 (0.0%)	2 (3.0%)	4 (2.1%)
No, I don't have any clinical experience with these patients but I would consult a cardio-oncology collegue if necessary	3 (4.5%)	0 (0.0%)	0 (0.0%)	8 (12.1%)	11 (5.8%)
22. In my hospital oncology and cardiology professionals easily contact eachother if needed					
missing	9 (13.6%)	6 (20.7%)	8 (27.6%)	26 (39.4%)	49 (25.8%)
Agree, we have a cardio-oncology multidisciplinary meeting	17 (25.8%)	1 (3.4%)	4 (13.8%)	1 (1.5%)	23 (12.1%)
Agree, oncologist consult cardiologists if there is a cancer patiënt that requires cardiac monitoring	30 (45.5%)	21 (72.4%)	15 (51.7%)	23 (34.8%)	89 (46.8%)
Disagree, no cardio-oncological care is performed. During active cancer treatment there is no focus on detecting potential cardiotoxicity	3 (4.5%)	1 (3.4%)	1 (3.4%)	11 (16.7%)	16 (8.4%)
Other, namely..	7 (10.6%)	0 (0.0%)	1 (3.4%)	5 (7.6%)	13 (6.8%)
	Other: no contact, only research contact, it should be a part of the healthcare trajectory to improve contact and reduce waitlist, currently working on setting up a cardio-oncology collaboration, referral only on indication				
23. Should cardiac monitoring of cancer patients be a priority in you opinion? (Select all that apply)					
Yes, early detection of cardiotoxicity is relevant for long-term cancer and cardiovascular health	43 (65.2%)	13 (44.8%)	11 (37.9%)	28 (42.4%)	95 (50.0%)
Yes, but it is the responsibility of cardiologists to diagnose and treat cardiotoxicity in cancer patients in a timely manner	11 (16.7%)	1 (3.4%)	2 (6.9%)	4 (6.1%)	18 (9.5%)
Yes, but it is the responsibility of oncologists to refer cancer patients with symptoms of cardiotoxicity	24 (36.4%)	11 (37.9%)	9 (31.0%)	16 (24.2%)	60 (31.6%)
No, morbidity and mortality related to cardiotoxicity of cancer therapy remains unclear	2 (3.0%)	3 (10.3%)	3 (10.3%)	2 (3.0%)	10 (5.3%)
No, for cancer patients potential cardiotoxicity of cancer therapy is not a priority	2 (3.0%)	0 (0.0%)	0 (0.0%)	0 (0.0%)	2 (1.1%)
24. What is needed to improve cardio-oncological care in your hospital?					
Dedicated cardio-oncologist	14 (21.2%)	11 (37.9%)	7 (24.1%)	17 (25.8%)	49 (25.8%)
Multidisciplinary meeting cardio-oncology	27 (40.9%)	2 (6.9%)	5 (17.2%)	4 (6.1%)	38 (20.0%)
More imaging capacity (Especially echocardiography and CMR)	24 (36.4%)	5 (17.2%)	7 (24.1%)	0 (0.0%)	36 (18.9%)
Nurse practitioner or physician assistant cardio-oncology	18 (27.3%)	4 (13.8%)	5 (17.2%)	6 (9.1%)	33 (17.4%)
Risk stratification algorithm	18 (27.3%)	7 (24.1%)	11 (37.9%)	24 (36.4%)	60 (31.6%)
Local protocol for cardiac monitoring and treatment of cardiotoxicity	20 (30.3%)	7 (24.1%)	15 (51.7%)	24 (36.4%)	66 (34.7%)
None of the above, cardiac surveillance is structurally perfomed in our hospital according to recent guidelines	12 (18.2%)	1 (3.4%)	0 (0.0%)	1 (1.5%)	14 (7.4%)
25. What is needed in the near future to improve cardio-oncological care in the Netherlands?					
Missing	11 (16.7%)	9 (31.0%)	8 (27.6%)	28 (42.4%)	56 (29.5%)
Cardio-oncology training	20 (30.3%)	5 (17.2%)	3 (10.3%)	10 (15.2%)	38 (20.0%)
Fellowship, including certification	2 (3.0%)	1 (3.4%)	0 (0.0%)	0 (0.0%)	3 (1.6%)
National guideline	29 (43.9%)	14 (48.3%)	17 (58.6%)	27 (40.9%)	87 (45.8%)
Other, namely	4 (6.1%)	0 (0.0%)	1 (3.4%)	1 (1.5%)	6 (3.2%)
	Other: Awareness, network, education				
